# TAPSE-to-tricuspid regurgitant pressure gradient ratio and early mortality in hospitalized heart failure

**DOI:** 10.1016/j.ahjo.2026.100876

**Published:** 2026-09-02

**Authors:** Isabelle Moseley, Akhil Vaid

**Affiliations:** a Department of Internal Medicine, Mount Sinai Morningside-West, New York, NY, USA; b Windreich Department of AI and Human Health, Icahn School of Medicine at Mount Sinai, New York, NY, USA

**Keywords:** Heart failure, Echocardiography, Right ventricle, TAPSE, Tricuspid regurgitation, Mortality

## Abstract

**Background:**

The TAPSE-to-TRPG ratio may summarize right-ventricular function relative to Doppler pressure load, but selective measurement and significant tricuspid regurgitation (TR) may limit its value.

**Methods:**

In linked MIMIC-IV and MIMIC-IV-ECHO data, we studied first heart-failure admissions with early echocardiography. Complete-case, inverse-probability-weighted, and multiply imputed models evaluated in-hospital and 30-day mortality; incremental performance, TR, cardiac index, timing, and competing discharge were examined.

**Results:**

Of 16,240 eligible patients, 2356 had TAPSE, TRPG, and numeric LVEF. Lower ratio was associated with in-hospital (OR 1.45, 95% CI 1.19–1.76) and 30-day mortality (OR 1.55, 95% CI 1.30–1.85), with consistent sensitivity estimates. TAPSE alone and separate-component models had greater cross-validated discrimination. The in-hospital association was not statistically evident in moderate-or-greater TR.

**Conclusions:**

Lower ratio identified higher early mortality but did not outperform its components; it remains hypothesis-generating rather than a validated incremental metric.

## Highlights

• Lower TAPSE-to-TRPG ratio was associated with early mortality.
• Associations persisted after missing-data and timing analyses.
• The ratio did not outperform TAPSE alone or separate components.
• In significant TR, the in-hospital association was not statistically evident.
• The ratio remains a hypothesis-generating structured-echo marker.

## 1 Introduction

Heart-failure prognosis is influenced not only by left ventricular ejection fraction (LVEF), but also by the ability of the right ventricle to adapt to pulmonary vascular and left-sided filling-pressure load. TAPSE is a routinely reported measure of longitudinal right-ventricular systolic function, while TRPG represents the Doppler-derived systolic pressure difference between the right ventricle and right atrium. Prior studies support TAPSE/pulmonary artery systolic pressure as a prognostic marker of right ventricular-pulmonary arterial coupling, including in acute heart failure [1], [2], [3], [4]. A TAPSE-to-TRPG ratio can be calculated from structured echocardiographic data when estimated right atrial pressure and pulmonary artery systolic pressure are unavailable, but it is not physiologically equivalent to TAPSE/pulmonary artery systolic pressure.

The validity of this pragmatic ratio may be limited by selective TAPSE and TRPG reporting, pressure equalization in severe TR, load dependence of TAPSE, and heterogeneous forward-flow states. Recent data in moderate and severe TR demonstrate adverse outcomes at both low and high cardiac index, suggesting that a single pressure-function ratio may not capture the full hemodynamic phenotype [5]. We therefore evaluated the association of the TAPSE-to-TRPG ratio with early mortality, formally addressed missingness and selection, compared the ratio with its components, and examined TR severity, cardiac index, LVEF phenotype, echo timing, and competing live discharge.

## 2 Methods

We performed a retrospective cohort study using MIMIC-IV v3.1 linked to MIMIC-IV-ECHO v1.0 [6], [7], [8], [9]. Adults with a hospital admission carrying an ICD-9 428.x or ICD-10 I50.x heart-failure diagnosis and a transthoracic echocardiogram from 24 h before through 48 h after admission were eligible; the first eligible admission per patient was retained. The exposure index was the later of admission or echocardiography, and patients who died or were discharged before that time were excluded. The TAPSE-to-TRPG ratio was calculated as TAPSE (cm) × 10/TRPG (mmHg). The primary outcome was in-hospital death after the exposure index; the secondary outcome was death within 30 days after the exposure index, ascertained from the MIMIC date-of-death field derived from hospital and state records. The source MIMIC projects were approved by the Beth Israel Deaconess Medical Center and Massachusetts Institute of Technology institutional review boards with waiver of individual informed consent; this secondary analysis used deidentified data under the PhysioNet data-use agreement.

The primary complete-case model included patients with TAPSE, TRPG, and numeric LVEF. The exposure was modeled continuously per 1-SD lower ratio; quartiles were descriptive. A causal diagram guided adjustment for age, sex, race, emergency admission, LVEF, atrial fibrillation, coronary artery disease, chronic kidney disease, chronic obstructive pulmonary disease, diabetes, prior cardiac surgery, creatinine, sodium, hemoglobin, and blood urea nitrogen. ICU admission, sepsis, and pulmonary-hypertension coding were treated as possible mediators or consequences and entered only in a sensitivity model.

We compared complete and excluded patients using standardized mean differences, modeled complete-case inclusion, and applied stabilized inverse-probability weights trimmed at the 1st and 99th percentiles. Twenty chained-equation multiple imputations with posterior draws included outcomes, clinical variables, TR severity, echo timing, cardiac index, and pre-echo intravenous loop diuretic, vasodilator, inotrope, and vasopressor exposure; the ratio was passively recalculated. Missing-not-at-random sensitivity analyses shifted imputed ratios 20% in either direction.

Identical-sample models compared the clinical model alone with addition of the ratio, TAPSE alone, TRPG alone, or TAPSE and TRPG separately using likelihood-ratio tests, information criteria, five-fold cross-validated area under the curve (AUC), Brier score, calibration, continuous net reclassification improvement (NRI), and integrated discrimination improvement (IDI). Additional analyses incorporated ordinal TR grade; stratified by moderate-or-greater TR, severe TR, atrial fibrillation, prior cardiac surgery, LVEF phenotype, and echo timing; used restricted cubic splines; and performed a 48-hour landmark analysis. Cardiac index was derived as [π(LVOT diameter / 2)^2^ × LVOT velocity-time integral × heart rate] / (1000 × body surface area) and categorized as low (<2.2), normal (2.2–4.0), or high (>4.0 L/min/m^2^). A cause-specific Cox model treated live discharge as a competing event. Subgroup, interaction, spline, NRI, and IDI analyses were exploratory; no multiplicity adjustment was applied. Two-sided *P* < 0.05 was considered statistically significant. Reporting followed STROBE and RECORD.

## 3 Results

**Fig. 1:**
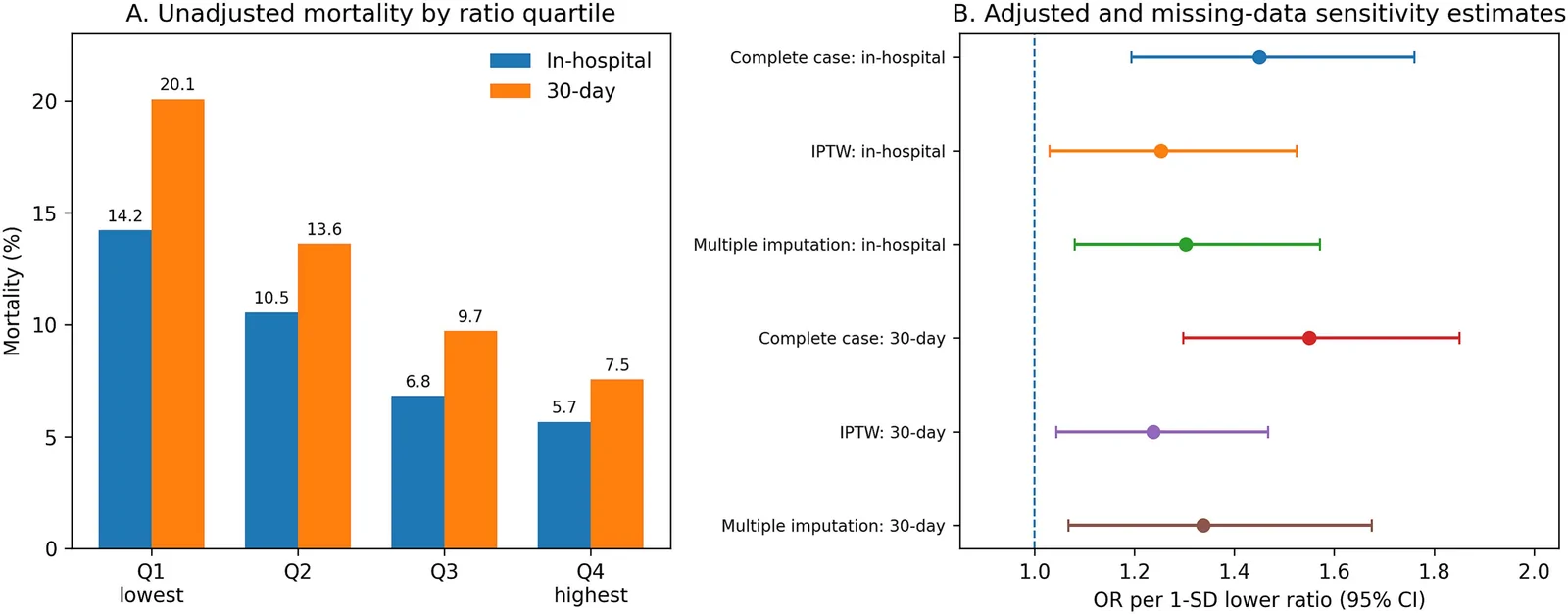
TAPSE-to-TRPG ratio and early mortality. Panel A shows unadjusted in-hospital and 30-day mortality across ratio quartiles. Panel B shows adjusted estimates per 1-SD lower ratio from complete-case, inverse-probability-weighted, and multiple-imputation analyses. CI indicates confidence interval; OR, odds ratio; TAPSE, tricuspid annular plane systolic excursion; TRPG, tricuspid regurgitant pressure gradient.

Among 80,611 heart-failure admissions, 22,070 had early echocardiography and 16,255 were first eligible admissions; 16,240 remained after exposure-index exclusions. TAPSE was available in 3094 patients, TRPG in 12,573, both in 2684, and TAPSE, TRPG, and numeric LVEF in 2356 (14.5%). The complete cohort included 220 in-hospital deaths (9.3%) and 301 30-day deaths (12.8%). The primary models contained 17 estimated predictor terms, corresponding to 12.9 and 17.7 events per term for in-hospital and 30-day mortality, respectively; both models converged without complete separation. Mortality declined across increasing ratio quartiles from 14.2% to 5.7% in hospital and from 20.1% to 7.5% by 30 days (Table 1; Fig. 1).

**Table 1:** Baseline characteristics by TAPSE-to-TRPG ratio quartile.

Characteristic	Overall	Q1 lowest	Q2	Q3	Q4 highest
Age, years	75.00 [65.00, 84.00]	78.00 [68.00, 85.75]	77.00 [68.00, 85.00]	74.00 [66.00, 82.00]	71.00 [62.00, 80.00]
Male sex	1278/2356 (54.2%)	316/598 (52.8%)	301/588 (51.2%)	312/587 (53.2%)	349/583 (59.9%)
LVEF, %	49.00 [34.00, 61.00]	45.00 [28.00, 60.00]	46.00 [31.75, 60.00]	50.00 [35.00, 62.00]	50.00 [40.00, 62.00]
TAPSE, cm	1.80 [1.40, 2.20]	1.30 [1.10, 1.60]	1.70 [1.40, 2.10]	1.90 [1.70, 2.20]	2.20 [1.90, 2.50]
TRPG, mmHg	34.00 [27.00, 44.00]	49.00 [41.00, 58.00]	38.00 [31.00, 44.00]	31.00 [27.00, 36.00]	23.00 [19.00, 27.00]
TAPSE-to-TRPG ratio	0.53 [0.37, 0.74]	0.29 [0.23, 0.33]	0.45 [0.41, 0.48]	0.62 [0.58, 0.68]	0.94 [0.81, 1.11]
Moderate-or-greater TR	738/2342 (31.5%)	369/597 (61.8%)	208/586 (35.5%)	108/582 (18.6%)	53/577 (9.2%)
Severe-or-greater TR	349/2342 (14.9%)	203/597 (34.0%)	78/586 (13.3%)	42/582 (7.2%)	26/577 (4.5%)
Cardiac index, L/min/m^2^	2.41 [1.91, 3.07]	2.20 [1.72, 2.81]	2.39 [1.88, 3.07]	2.49 [2.02, 3.19]	2.55 [1.99, 3.11]
Atrial fibrillation	1218/2356 (51.7%)	384/598 (64.2%)	346/588 (58.8%)	267/587 (45.5%)	221/583 (37.9%)
Chronic kidney disease	964/2356 (40.9%)	274/598 (45.8%)	259/588 (44.0%)	230/587 (39.2%)	201/583 (34.5%)
Pulmonary hypertension code	603/2356 (25.6%)	277/598 (46.3%)	177/588 (30.1%)	106/587 (18.1%)	43/583 (7.4%)
Prior cardiac surgery	330/2356 (14.0%)	124/598 (20.7%)	86/588 (14.6%)	73/587 (12.4%)	47/583 (8.1%)
Creatinine, mg/dL	1.20 [0.90, 1.70]	1.30 [1.00, 1.90]	1.20 [0.90, 1.90]	1.10 [0.90, 1.70]	1.10 [0.85, 1.50]
BUN, mg/dL	24.00 [17.00, 39.00]	29.00 [19.00, 46.00]	27.00 [18.00, 41.00]	22.00 [16.00, 33.00]	21.00 [15.00, 33.00]
Any pre-echo hemodynamic treatment	1116/2356 (47.4%)	319/598 (53.3%)	310/588 (52.7%)	282/587 (48.0%)	205/583 (35.2%)
In-hospital death after exposure	220/2356 (9.3%)	85/598 (14.2%)	62/588 (10.5%)	40/587 (6.8%)	33/583 (5.7%)
30-day death after exposure	301/2356 (12.8%)	120/598 (20.1%)	80/588 (13.6%)	57/587 (9.7%)	44/583 (7.5%)

Values are median [interquartile range] or n/N (%). Cardiac index was LVOT-derived. TR indicates tricuspid regurgitation.

Per 1-SD lower ratio, adjusted ORs were 1.45 (95% CI 1.19–1.76; *P* < 0.001) for in-hospital death and 1.55 (95% CI 1.30–1.85; *P* < 0.001) for 30-day death. Estimates persisted with selection weighting (OR 1.25 and 1.24), multiple imputation (OR 1.30 and 1.34), a 48-hour landmark (OR 1.33 and 1.42), and a cause-specific competing-discharge model (hazard ratio 1.26 for in-hospital death). Formal comparison confirmed marked selection into the complete cohort; the selection model AUC was 0.793.

The ratio improved likelihood fit over the clinical model, but did not outperform its components. Cross-validated AUCs for in-hospital death were 0.659 for the clinical model, 0.673 with the ratio, 0.686 with TAPSE alone, and 0.685 with TAPSE and TRPG separately; corresponding 30-day AUCs were 0.668, 0.684, 0.699, and 0.700 (Table 2). Moderate-or-greater TR was present in 31.5%. The in-hospital association was evident without moderate-or-greater TR (OR 1.51) but was not statistically evident with it (OR 1.02; interaction *P* = 0.052); the borderline interaction should be interpreted cautiously. The 30-day association remained in both strata. Cardiac index was available in 91.5%; adjusted low-flow status was associated with both outcomes and high-flow status with 30-day mortality, with an exploratory ratio-by-flow interaction for 30-day mortality (*P* = 0.015). LVEF-phenotype interactions were not significant.

**Table 2:** Association and cross-validated model performance.

Outcome	Added echo measure(s)	Adjusted OR (95% CI)	CV AUC	CV Brier	Calibration slope
In-hospital death	TAPSE-to-TRPG ratio	1.45 (1.19–1.76)	0.673	0.083	0.75
In-hospital death	TAPSE alone	1.60 (1.35–1.90)	0.686	0.082	0.79
In-hospital death	TRPG alone	1.13 (0.98–1.31)	0.660	0.083	0.72
In-hospital death	TAPSE and TRPG separate	TAPSE: 1.59 (1.34–1.88); TRPG: 1.08 (0.94–1.24)	0.685	0.082	0.77
30-day death	TAPSE-to-TRPG ratio	1.55 (1.30–1.85)	0.684	0.106	0.80
30-day death	TAPSE alone	1.65 (1.42–1.92)	0.699	0.105	0.82
30-day death	TRPG alone	1.23 (1.09–1.40)	0.674	0.107	0.79
30-day death	TAPSE and TRPG separate	TAPSE: 1.61 (1.38–1.88); TRPG: 1.17 (1.04–1.33)	0.700	0.105	0.82

Odds ratios are per 1-SD higher-risk direction: lower ratio, lower TAPSE, or higher TRPG. Models used the identical complete-case sample and the same clinical covariates. CV indicates five-fold cross-validation.

## 4 Discussion

In hospitalized patients with available TAPSE and TRPG, a lower TAPSE-to-TRPG ratio was consistently associated with early mortality across complete-case, multiple-imputation, selection-weighted, timing, landmark, and competing-discharge analyses. The revised analyses also materially narrow the interpretation. First, measurement availability was strongly non-random, and sensitivity methods attenuated the estimates. Second, the ratio did not outperform TAPSE alone or TAPSE and TRPG entered separately. Thus, these data demonstrate association, not superiority or validated incremental clinical utility.

TR severity is mechanistically important. In severe TR, right ventricular-right atrial systolic pressure equalization may lower TRPG despite advanced disease, while TAPSE remains load dependent. The in-hospital association was attenuated and not statistically evident in moderate-or-greater TR, although the interaction test was borderline (*P* = 0.052). This finding supports caution in interpreting the ratio as a uniform marker of right ventricular-pulmonary arterial coupling. Atrial fibrillation and prior surgery did not show significant interactions, although the surgery subgroup was small.

Forward-flow profiling added complementary information. Both low- and high-cardiac-index groups had higher unadjusted mortality than the intermediate group, consistent with hypodynamic-uncoupled and hyperdynamic-congestive phenotypes described in significant TR [5]. The adjusted spline did not establish a continuous U-shaped relationship, but low flow remained associated with both outcomes and high flow with 30-day death. Future risk models should integrate pressure-function measures with forward flow and multiorgan congestion markers, including lung, inferior vena cava, and venous Doppler assessments, which can provide incremental prognostic information but were unavailable in MIMIC-IV-ECHO [10]. These subgroup and interaction findings are exploratory and require external validation.

Limitations include single-center retrospective data, selective echo measurement, residual and unmeasured confounding, possible treatment-related alteration of TRPG within the 72-hour echo window, and absence of invasive hemodynamics, explicit right atrial pressure, adjudicated heart-failure etiology, and multiorgan ultrasound. Post-discharge mortality was derived from linked records rather than independent adjudication. Even extensive imputation cannot exclude missing-not-at-random bias when approximately 81% of TAPSE values are absent.

## 5 Conclusions

Among hospitalized heart-failure patients with measurable TAPSE and TRPG, a lower TAPSE-to-TRPG ratio was associated with greater in-hospital and 30-day mortality. The ratio did not outperform TAPSE alone or its components entered separately. In moderate-or-greater TR, the in-hospital association was attenuated and not statistically evident, with a borderline interaction. These findings support the ratio as a hypothesis-generating structured-echo marker, not a validated incremental risk metric.

## CRediT authorship contribution statement

**Isabelle Moseley:** Writing – original draft, Formal analysis, Data curation, Conceptualization. **Akhil Vaid:** Writing – review & editing, Supervision, Methodology, Investigation, Formal analysis.

## Ethics approval

The source MIMIC projects were approved by the Beth Israel Deaconess Medical Center and Massachusetts Institute of Technology institutional review boards with waiver of individual informed consent. This secondary analysis used deidentified data under the PhysioNet data-use agreement.

## Ethics statement

The MIMIC resources are deidentified. The source MIMIC-IV-ECHO project was approved by the Institutional Review Boards of Beth Israel Deaconess Medical Center (Boston, MA) and the Massachusetts Institute of Technology (Cambridge, MA), with waiver of individual informed consent because the project did not affect clinical care and all protected health information was deidentified [8]. The present analysis used only deidentified data accessed by credentialed users under the applicable PhysioNet data use agreement and involved no patient contact, intervention, experimentation, organ or tissue use, or access to identifiable private information. All procedures were conducted in accordance with relevant laws, institutional guidelines, and the principles of the Declaration of Helsinki as applicable to secondary analysis of deidentified clinical data. Privacy rights of human subjects were protected through deidentification, credentialed access, required training, and data-use restrictions. No additional informed consent was required for this secondary analysis of deidentified data.

## Declaration of Generative AI and AI-assisted technologies in the writing process

During manuscript preparation, the authors used OpenAI's ChatGPT for organization, formatting, journal-guideline checks, and language editing. The authors reviewed and edited all content and take full responsibility for the manuscript.

## Funding

This research did not receive any specific grant from funding agencies in the public, commercial, or not-for-profit sectors.

## Declaration of competing interest

The authors declare no known competing financial interests or personal relationships that could have influenced the work reported in this paper.

## Data Availability

MIMIC-IV and MIMIC-IV-ECHO are available to credentialed PhysioNet users under their data-use agreements. The complete analytic code and aggregate analysis outputs are supplied with the revision.
