# Supplementary material for: TAPSE-to-tricuspid regurgitant pressure gradient ratio and early mortality in hospitalized heart failure
Source: Am Heart J Plus. 2026 Sep 2;70:100876. doi: 10.1016/j.ahjo.2026.100876 (PMC13571287; doi:10.1016/j.ahjo.2026.100876)
Supplement: Supplementary file 1 [file mmc1.docx]

Supplementary Material

TAPSE-to-tricuspid regurgitant pressure gradient ratio and early mortality in hospitalized heart failure

Isabelle Moseley, MD; Akhil Vaid, MD

# Supplementary Methods

## Data extraction and cohort assembly

Structured measurements were audited against the MIMIC-IV-ECHO measurement dictionary. The first heart-failure admission with transthoracic echocardiography from 24 hours before through 48 hours after admission was retained. Exposure ascertainment was anchored at the later of hospital admission or echocardiography; patients dead or discharged before this time were excluded. Exact numeric LVEF fields were used, explaining the revised complete-case count.

## Exposure and outcomes

The ratio was TAPSE (cm) × 10/TRPG (mmHg). For regression, the ratio was standardized so odds ratios represent a 1-SD lower value (complete-case SD 0.321). TAPSE was similarly oriented so higher values indicate lower function, and TRPG so higher values indicate higher gradient. Death within 30 days was measured from the exposure index using the MIMIC date-of-death field.

## Primary covariate set

The confounder model included age/10 years, sex, White race, emergency admission, LVEF/10%, atrial fibrillation, coronary artery disease, chronic kidney disease, COPD, diabetes, prior cardiac surgery, log creatinine, sodium per 5 mmol/L, hemoglobin, BUN/10 mg/dL, and an indicator of any core laboratory missingness. ICU admission, sepsis, and pulmonary-hypertension coding were excluded from the primary set and added only in sensitivity analysis.

## Missing-data analyses

Included and excluded patients were compared with standardized mean differences. A logistic selection model estimated the probability of complete TAPSE, TRPG, and LVEF measurement; stabilized inverse-probability weights were trimmed at the 1st and 99th percentiles. Twenty chained-equation imputations used Bayesian ridge models with posterior sampling. The ratio was passively derived from imputed TAPSE and TRPG. Bidirectional delta analyses shifted missing TAPSE and TRPG to generate ratios 20% lower or higher than under missing at random.

## Incremental model comparison

Clinical, ratio, TAPSE, TRPG, and separate-component models were evaluated in the same 2,356 patients. Nested likelihood-ratio tests, AIC/BIC, five-fold cross-validated AUC, Brier score, calibration intercept and slope, continuous NRI, and IDI were calculated. Bootstrap confidence intervals used 200 resamples. The primary model contained 17 estimated predictor terms. With 220 in-hospital and 301 30-day deaths, this corresponded to 12.9 and 17.7 events per term, respectively; all primary models converged without complete separation.

## TR, flow, and timing analyses

TR grade was entered ordinally and used for stratification, exclusion, and interaction testing. Cardiac index was derived as [π(LVOT diameter/2)² × LVOT velocity-time integral × heart rate]/(1,000 × body surface area) and categorized as <2.2, 2.2-4.0, or >4.0 L/min/m². Restricted cubic splines used four knots. The 48-hour landmark analysis included patients alive and hospitalized after the complete echo window. The cause-specific Cox model censored live discharge. Subgroup, interaction, spline, NRI, and IDI analyses were exploratory; no multiplicity adjustment was applied, and P values should be interpreted accordingly.

## Reproducibility

The SQL extraction scripts, Python analysis scripts, aggregate tables, and figures are supplied with the revision. Analyses used Python 3.11/3.13 with pandas, NumPy, statsmodels, scikit-learn, patsy, and lifelines/statistical survival routines as specified in the included environment files.

**Table S1. Cohort flow and variable coverage**

| **Stage** | **N** |
| --- | --- |
| HF admissions in original extraction | 80,611 |
| Admissions with early TTE in original extraction | 22,070 |
| First eligible HF admissions | 16,255 |
| Eligible after excluding death/discharge before exposure | 16,240 |
| TAPSE observed | 3,094 |
| TRPG observed | 12,573 |
| TAPSE and TRPG observed | 2,684 |
| TAPSE, TRPG, and numeric LVEF observed | 2,356 |
| Complete cohort with cardiac index | 2,155 |

| **Variable** | **Full eligible cohort** | **Complete cohort** |
| --- | --- | --- |
| tapse_cm | 3,094 (19.1%) | 2,356 (100.0%) |
| trpg_mmhg | 12,573 (77.4%) | 2,356 (100.0%) |
| tapse_to_trpg_ratio | 2,684 (16.5%) | 2,356 (100.0%) |
| lvef_percent | 15,193 (93.6%) | 2,356 (100.0%) |
| tr_grade_numeric | 14,423 (88.8%) | 2,342 (99.4%) |
| significant_tr_at_least_moderate | 14,423 (88.8%) | 2,342 (99.4%) |
| severe_or_greater_tr | 14,423 (88.8%) | 2,342 (99.4%) |
| pasp_underestimated_due_to_severe_tr | 16,240 (100.0%) | 2,356 (100.0%) |
| cardiac_index_best_l_min_m2 | 10,273 (63.3%) | 2,155 (91.5%) |
| prior_cardiac_surgery | 16,240 (100.0%) | 2,356 (100.0%) |
| echo_hours_from_admission | 16,240 (100.0%) | 2,356 (100.0%) |
| pre_echo_iv_loop_diuretic | 16,240 (100.0%) | 2,356 (100.0%) |
| pre_echo_iv_vasodilator | 16,240 (100.0%) | 2,356 (100.0%) |
| pre_echo_inotrope | 16,240 (100.0%) | 2,356 (100.0%) |
| pre_echo_vasopressor | 16,240 (100.0%) | 2,356 (100.0%) |

**Table S2. Complete-case versus excluded cohort**

| **Variable** | **Complete cases** | **Excluded** | **SMD** | **\|SMD\|** |
| --- | --- | --- | --- | --- |
| emergency_admission | 880/2356 (37.4%) | 8674/13884 (62.5%) | -0.502 | 0.502 |
| any_requested_pre_echo_hemodynamic_treatment | 1116/2356 (47.4%) | 4804/13884 (34.6%) | 0.260 | 0.260 |
| pulmonary_hypertension_code | 603/2356 (25.6%) | 2619/13884 (18.9%) | 0.162 | 0.162 |
| significant_tr_at_least_moderate | 738/2342 (31.5%) | 3020/12081 (25.0%) | 0.145 | 0.145 |
| white_race | 1491/2356 (63.3%) | 9699/13884 (69.9%) | -0.139 | 0.139 |
| prior_cardiac_surgery | 330/2356 (14.0%) | 2528/13884 (18.2%) | -0.114 | 0.114 |
| atrial_fibrillation | 1218/2356 (51.7%) | 6410/13884 (46.2%) | 0.111 | 0.111 |
| cardiac_index_best_l_min_m2 | 2.41 [1.91, 3.07] | 2.51 [2.01, 3.16] | -0.108 | 0.108 |
| lvef_percent | 49.00 [34.00, 61.00] | 50.00 [31.00, 60.00] | 0.074 | 0.074 |
| admission_hemoglobin | 11.00 [9.20, 12.70] | 11.10 [9.60, 12.60] | -0.072 | 0.072 |
| chronic_kidney_disease | 964/2356 (40.9%) | 5202/13884 (37.5%) | 0.071 | 0.071 |
| sepsis_code | 306/2356 (13.0%) | 1508/13884 (10.9%) | 0.066 | 0.066 |
| inhospital_death_after_exposure | 220/2356 (9.3%) | 1078/13884 (7.8%) | 0.056 | 0.056 |
| death_30d_after_exposure | 301/2356 (12.8%) | 1576/13884 (11.4%) | 0.044 | 0.044 |
| admission_bun | 24.00 [17.00, 39.00] | 25.00 [17.00, 40.00] | -0.043 | 0.043 |
| coronary_artery_disease | 1348/2356 (57.2%) | 8209/13884 (59.1%) | -0.039 | 0.039 |
| age | 75.00 [65.00, 84.00] | 75.00 [64.00, 84.00] | 0.035 | 0.035 |
| copd | 621/2356 (26.4%) | 3855/13884 (27.8%) | -0.032 | 0.032 |
| admission_creatinine | 1.20 [0.90, 1.70] | 1.20 [0.90, 1.80] | 0.030 | 0.030 |
| diabetes | 955/2356 (40.5%) | 5789/13884 (41.7%) | -0.024 | 0.024 |
| icu_before_or_at_echo | 824/2356 (35.0%) | 4766/13884 (34.3%) | 0.014 | 0.014 |
| echo_hours_from_admission | 17.04 [10.78, 30.26] | 17.68 [11.33, 27.80] | -0.011 | 0.011 |
| admission_sodium | 139.00 [136.00, 142.00] | 139.00 [136.00, 141.00] | -0.010 | 0.010 |
| male | 1278/2356 (54.2%) | 7563/13884 (54.5%) | -0.005 | 0.005 |

SMD indicates standardized mean difference. Absolute SMD ≥0.10 was considered a meaningful imbalance.

**Table S3. Primary exposure-component associations and likelihood-ratio tests**

| **Outcome** | **Analysis** | **Model** | **Term** | **Estimate (95% CI) or χ²** | **P** |
| --- | --- | --- | --- | --- | --- |
| In-hospital death after exposure | Complete-case logistic regression | TAPSE-to-TRPG ratio | tapse_to_trpg_ratio_riskz | 1.45 (1.19-1.76) | <0.001 |
| In-hospital death after exposure | Complete-case logistic regression | TAPSE alone | tapse_cm_riskz | 1.60 (1.35-1.90) | <0.001 |
| In-hospital death after exposure | Complete-case logistic regression | TRPG alone | trpg_mmhg_riskz | 1.13 (0.98-1.31) | 0.085 |
| In-hospital death after exposure | Complete-case logistic regression | TAPSE and TRPG separate | tapse_cm_riskz | 1.59 (1.34-1.88) | <0.001 |
| In-hospital death after exposure | Complete-case logistic regression | TAPSE and TRPG separate | trpg_mmhg_riskz | 1.08 (0.94-1.24) | 0.288 |
| In-hospital death after exposure | Complete-case logistic regression | Ratio plus possible mediators | tapse_to_trpg_ratio_riskz | 1.47 (1.18-1.83) | <0.001 |
| In-hospital death after exposure | Complete-case logistic regression | Lowest ratio quartile vs Q2-Q4 | lowest_ratio_q | 1.71 (1.26-2.33) | <0.001 |
| In-hospital death after exposure | Likelihood-ratio test vs clinical model | TAPSE-to-TRPG ratio | Added exposure term(s) | 15.92 | <0.001 |
| In-hospital death after exposure | Likelihood-ratio test vs clinical model | TAPSE alone | Added exposure term(s) | 30.91 | <0.001 |
| In-hospital death after exposure | Likelihood-ratio test vs clinical model | TRPG alone | Added exposure term(s) | 2.89 | 0.089 |
| In-hospital death after exposure | Likelihood-ratio test vs clinical model | TAPSE and TRPG separate | Added exposure term(s) | 32.02 | <0.001 |
| 30-day death after exposure | Complete-case logistic regression | TAPSE-to-TRPG ratio | tapse_to_trpg_ratio_riskz | 1.55 (1.30-1.85) | <0.001 |
| 30-day death after exposure | Complete-case logistic regression | TAPSE alone | tapse_cm_riskz | 1.65 (1.42-1.92) | <0.001 |
| 30-day death after exposure | Complete-case logistic regression | TRPG alone | trpg_mmhg_riskz | 1.23 (1.09-1.40) | <0.001 |
| 30-day death after exposure | Complete-case logistic regression | TAPSE and TRPG separate | tapse_cm_riskz | 1.61 (1.38-1.88) | <0.001 |
| 30-day death after exposure | Complete-case logistic regression | TAPSE and TRPG separate | trpg_mmhg_riskz | 1.17 (1.04-1.33) | 0.012 |
| 30-day death after exposure | Complete-case logistic regression | Ratio plus possible mediators | tapse_to_trpg_ratio_riskz | 1.57 (1.29-1.90) | <0.001 |
| 30-day death after exposure | Complete-case logistic regression | Lowest ratio quartile vs Q2-Q4 | lowest_ratio_q | 1.77 (1.35-2.33) | <0.001 |
| 30-day death after exposure | Likelihood-ratio test vs clinical model | TAPSE-to-TRPG ratio | Added exposure term(s) | 26.84 | <0.001 |
| 30-day death after exposure | Likelihood-ratio test vs clinical model | TAPSE alone | Added exposure term(s) | 43.63 | <0.001 |
| 30-day death after exposure | Likelihood-ratio test vs clinical model | TRPG alone | Added exposure term(s) | 10.54 | 0.001 |
| 30-day death after exposure | Likelihood-ratio test vs clinical model | TAPSE and TRPG separate | Added exposure term(s) | 49.80 | <0.001 |

**Table S4. Cross-validated performance and incremental metrics**

| **Outcome** | **Model** | **CV AUC** | **CV Brier** | **Cal intercept** | **Cal slope** | **AIC** | **BIC** |
| --- | --- | --- | --- | --- | --- | --- | --- |
| In-hospital death after exposure | Clinical model only | 0.659 | 0.083 | -0.004 | 0.733 | 1400.1 | 1498.1 |
| In-hospital death after exposure | TAPSE-to-TRPG ratio | 0.673 | 0.083 | -0.004 | 0.753 | 1386.2 | 1490.0 |
| In-hospital death after exposure | TAPSE alone | 0.686 | 0.082 | -0.003 | 0.785 | 1371.2 | 1475.0 |
| In-hospital death after exposure | TRPG alone | 0.660 | 0.083 | -0.004 | 0.725 | 1399.2 | 1503.0 |
| In-hospital death after exposure | TAPSE and TRPG separate | 0.685 | 0.082 | -0.003 | 0.770 | 1372.1 | 1481.6 |
| 30-day death after exposure | Clinical model only | 0.668 | 0.107 | -0.004 | 0.791 | 1695.1 | 1793.1 |
| 30-day death after exposure | TAPSE-to-TRPG ratio | 0.684 | 0.106 | -0.004 | 0.798 | 1670.3 | 1774.0 |
| 30-day death after exposure | TAPSE alone | 0.699 | 0.105 | -0.004 | 0.825 | 1653.5 | 1757.2 |
| 30-day death after exposure | TRPG alone | 0.674 | 0.107 | -0.004 | 0.792 | 1686.6 | 1790.3 |
| 30-day death after exposure | TAPSE and TRPG separate | 0.700 | 0.105 | -0.005 | 0.818 | 1649.3 | 1758.8 |

| **Outcome** | **Comparison** | **ΔAUC (95% CI)** | **Continuous NRI (95% CI)** | **IDI (95% CI)** | **ΔBrier** |
| --- | --- | --- | --- | --- | --- |
| In-hospital death after exposure | TAPSE-to-TRPG ratio vs clinical model | 0.015 (-0.001 to 0.032) | 0.261 (0.137 to 0.394) | 0.006 (0.001 to 0.011) | -0.0005 |
| In-hospital death after exposure | TAPSE alone vs clinical model | 0.027 (0.007 to 0.047) | 0.293 (0.151 to 0.425) | 0.017 (0.009 to 0.024) | -0.0015 |
| In-hospital death after exposure | TRPG alone vs clinical model | 0.001 (-0.006 to 0.008) | 0.155 (0.007 to 0.298) | 0.000 (-0.002 to 0.002) | 0.0001 |
| In-hospital death after exposure | TAPSE and TRPG separate vs clinical model | 0.027 (0.005 to 0.049) | 0.288 (0.158 to 0.439) | 0.016 (0.008 to 0.023) | -0.0012 |
| In-hospital death after exposure | TAPSE and TRPG separate vs ratio | 0.012 (-0.004 to 0.027) | 0.231 (0.104 to 0.356) | 0.009 (0.004 to 0.015) | -0.0007 |
| 30-day death after exposure | TAPSE-to-TRPG ratio vs clinical model | 0.016 (0.001 to 0.029) | 0.259 (0.148 to 0.379) | 0.009 (0.003 to 0.015) | -0.0007 |
| 30-day death after exposure | TAPSE alone vs clinical model | 0.030 (0.012 to 0.048) | 0.314 (0.184 to 0.432) | 0.019 (0.012 to 0.026) | -0.0018 |
| 30-day death after exposure | TRPG alone vs clinical model | 0.005 (-0.006 to 0.015) | 0.140 (0.031 to 0.254) | 0.004 (0.000 to 0.009) | -0.0002 |
| 30-day death after exposure | TAPSE and TRPG separate vs clinical model | 0.031 (0.011 to 0.050) | 0.344 (0.247 to 0.452) | 0.022 (0.014 to 0.030) | -0.0019 |
| 30-day death after exposure | TAPSE and TRPG separate vs ratio | 0.016 (0.004 to 0.028) | 0.253 (0.124 to 0.378) | 0.012 (0.007 to 0.018) | -0.0012 |

**Table S5. Missing-data sensitivity analyses**

| **Outcome** | **Analysis** | **OR (95% CI)** | **P** |
| --- | --- | --- | --- |
| In-hospital death after exposure | IPTW for complete-case selection (1st/99th trimmed) | 1.25 (1.03-1.53) | 0.024 |
| 30-day death after exposure | IPTW for complete-case selection (1st/99th trimmed) | 1.24 (1.04-1.47) | 0.014 |
| 30-day death after exposure | Multiple imputation: MAR | 1.34 (1.07-1.68) | 0.014 |
| In-hospital death after exposure | Multiple imputation: MAR | 1.30 (1.08-1.57) | 0.007 |
| 30-day death after exposure | Multiple imputation: Missing ratio 20% higher | 1.23 (1.04-1.45) | 0.018 |
| In-hospital death after exposure | Multiple imputation: Missing ratio 20% higher | 1.20 (1.04-1.39) | 0.012 |
| 30-day death after exposure | Multiple imputation: Missing ratio 20% lower | 1.43 (1.10-1.85) | 0.010 |
| In-hospital death after exposure | Multiple imputation: Missing ratio 20% lower | 1.38 (1.11-1.72) | 0.005 |

**Table S6. TR severity, atrial fibrillation, prior surgery, LVEF, and echo-timing analyses**

| **Outcome** | **Analysis** | **Subgroup** | **Modifier** | **Estimate** | **P** |
| --- | --- | --- | --- | --- | --- |
| In-hospital death after exposure | Primary confounder-only | All complete cases | Per 1-SD lower ratio | 1.45 (1.19-1.76) | <0.001 |
| In-hospital death after exposure | Original-style model including ICU/sepsis/pulmonary HTN | All complete cases | Per 1-SD lower ratio | 1.47 (1.18-1.83) | <0.001 |
| In-hospital death after exposure | Adjusted for ordinal TR grade | TR grade available | Per 1-SD lower ratio | 1.44 (1.16-1.78) | <0.001 |
| In-hospital death after exposure | Stratified sensitivity | No moderate-or-greater TR | Per 1-SD lower ratio | 1.51 (1.17-1.96) | 0.002 |
| In-hospital death after exposure | Stratified sensitivity | Moderate-or-greater TR | Per 1-SD lower ratio | 1.02 (0.69-1.49) | 0.935 |
| In-hospital death after exposure | Stratified sensitivity | Excluding severe-or-greater TR | Per 1-SD lower ratio | 1.48 (1.19-1.85) | <0.001 |
| In-hospital death after exposure | Stratified sensitivity | No atrial fibrillation | Per 1-SD lower ratio | 1.50 (1.13-1.99) | 0.005 |
| In-hospital death after exposure | Stratified sensitivity | Atrial fibrillation | Per 1-SD lower ratio | 1.37 (1.05-1.79) | 0.023 |
| In-hospital death after exposure | Stratified sensitivity | No prior cardiac surgery | Per 1-SD lower ratio | 1.49 (1.21-1.84) | <0.001 |
| In-hospital death after exposure | Stratified sensitivity | Prior cardiac surgery | Per 1-SD lower ratio | 1.28 (0.77-2.13) | 0.342 |
| In-hospital death after exposure | Stratified sensitivity | Echo <=24 hours | Per 1-SD lower ratio | 1.39 (1.12-1.74) | 0.003 |
| In-hospital death after exposure | Stratified sensitivity | Echo >24-48 hours | Per 1-SD lower ratio | 1.56 (1.03-2.36) | 0.035 |
| In-hospital death after exposure | Interaction test | All with modifier available | significant_tr_at_least_moderate | χ²=3.78 | 0.052 |
| In-hospital death after exposure | Interaction test | All with modifier available | severe_or_greater_tr | χ²=0.64 | 0.424 |
| In-hospital death after exposure | Interaction test | All with modifier available | atrial_fibrillation | χ²=0.16 | 0.688 |
| In-hospital death after exposure | Interaction test | All with modifier available | prior_cardiac_surgery | χ²=0.05 | 0.828 |
| In-hospital death after exposure | Interaction test | All with modifier available | late_echo | χ²=0.48 | 0.486 |
| In-hospital death after exposure | LVEF phenotype stratification | HFrEF <40% | Per 1-SD lower ratio | 1.19 (0.87-1.62) | 0.283 |
| In-hospital death after exposure | LVEF phenotype stratification | HFmrEF 40-49% | Per 1-SD lower ratio | 1.21 (0.72-2.02) | 0.475 |
| In-hospital death after exposure | LVEF phenotype stratification | HFpEF >=50% | Per 1-SD lower ratio | 1.59 (1.18-2.14) | 0.002 |
| In-hospital death after exposure | Joint LVEF phenotype interaction | All complete cases | Ratio x LVEF phenotype | χ²=0.90 | 0.638 |
| In-hospital death after exposure | Adjusted for echo timing | All complete cases | Per 1-SD lower ratio | 1.44 (1.19-1.75) | <0.001 |
| 30-day death after exposure | Primary confounder-only | All complete cases | Per 1-SD lower ratio | 1.55 (1.30-1.85) | <0.001 |
| 30-day death after exposure | Original-style model including ICU/sepsis/pulmonary HTN | All complete cases | Per 1-SD lower ratio | 1.57 (1.29-1.90) | <0.001 |
| 30-day death after exposure | Adjusted for ordinal TR grade | TR grade available | Per 1-SD lower ratio | 1.57 (1.29-1.91) | <0.001 |
| 30-day death after exposure | Stratified sensitivity | No moderate-or-greater TR | Per 1-SD lower ratio | 1.49 (1.19-1.86) | <0.001 |
| 30-day death after exposure | Stratified sensitivity | Moderate-or-greater TR | Per 1-SD lower ratio | 1.51 (1.03-2.21) | 0.036 |
| 30-day death after exposure | Stratified sensitivity | Excluding severe-or-greater TR | Per 1-SD lower ratio | 1.49 (1.23-1.81) | <0.001 |
| 30-day death after exposure | Stratified sensitivity | No atrial fibrillation | Per 1-SD lower ratio | 1.82 (1.38-2.40) | <0.001 |
| 30-day death after exposure | Stratified sensitivity | Atrial fibrillation | Per 1-SD lower ratio | 1.35 (1.07-1.71) | 0.012 |
| 30-day death after exposure | Stratified sensitivity | No prior cardiac surgery | Per 1-SD lower ratio | 1.62 (1.34-1.97) | <0.001 |
| 30-day death after exposure | Stratified sensitivity | Prior cardiac surgery | Per 1-SD lower ratio | 1.29 (0.83-2.02) | 0.262 |
| 30-day death after exposure | Stratified sensitivity | Echo <=24 hours | Per 1-SD lower ratio | 1.62 (1.31-2.00) | <0.001 |
| 30-day death after exposure | Stratified sensitivity | Echo >24-48 hours | Per 1-SD lower ratio | 1.30 (0.93-1.82) | 0.123 |
| 30-day death after exposure | Interaction test | All with modifier available | significant_tr_at_least_moderate | χ²=0.00 | 0.950 |
| 30-day death after exposure | Interaction test | All with modifier available | severe_or_greater_tr | χ²=1.76 | 0.184 |
| 30-day death after exposure | Interaction test | All with modifier available | atrial_fibrillation | χ²=2.38 | 0.123 |
| 30-day death after exposure | Interaction test | All with modifier available | prior_cardiac_surgery | χ²=0.30 | 0.584 |
| 30-day death after exposure | Interaction test | All with modifier available | late_echo | χ²=0.61 | 0.433 |
| 30-day death after exposure | LVEF phenotype stratification | HFrEF <40% | Per 1-SD lower ratio | 1.21 (0.92-1.61) | 0.174 |
| 30-day death after exposure | LVEF phenotype stratification | HFmrEF 40-49% | Per 1-SD lower ratio | 1.20 (0.81-1.79) | 0.368 |
| 30-day death after exposure | LVEF phenotype stratification | HFpEF >=50% | Per 1-SD lower ratio | 1.91 (1.43-2.55) | <0.001 |
| 30-day death after exposure | Joint LVEF phenotype interaction | All complete cases | Ratio x LVEF phenotype | χ²=3.43 | 0.180 |
| 30-day death after exposure | Adjusted for echo timing | All complete cases | Per 1-SD lower ratio | 1.54 (1.29-1.84) | <0.001 |

**Table S7. Cardiac-index analyses and joint flow/ratio profiles**

| **Outcome** | **Analysis** | **Term** | **Estimate** | **P** |
| --- | --- | --- | --- | --- |
| In-hospital death after exposure | Flow-state categorical model | tapse_to_trpg_ratio_riskz | 1.47 (1.19-1.82) | <0.001 |
| In-hospital death after exposure | Flow-state categorical model | flow_low | 1.82 (1.30-2.56) | <0.001 |
| In-hospital death after exposure | Flow-state categorical model | flow_high | 1.56 (0.88-2.77) | 0.129 |
| In-hospital death after exposure | Ratio-by-flow-state interaction | Joint interaction | χ²=4.60 | 0.100 |
| In-hospital death after exposure | Cardiac-index spline nonlinearity | Nonlinear vs linear cardiac index | χ²=1.54 | 0.672 |
| 30-day death after exposure | Flow-state categorical model | tapse_to_trpg_ratio_riskz | 1.62 (1.34-1.97) | <0.001 |
| 30-day death after exposure | Flow-state categorical model | flow_low | 1.45 (1.08-1.95) | 0.015 |
| 30-day death after exposure | Flow-state categorical model | flow_high | 1.71 (1.04-2.81) | 0.034 |
| 30-day death after exposure | Ratio-by-flow-state interaction | Joint interaction | χ²=8.39 | 0.015 |
| 30-day death after exposure | Cardiac-index spline nonlinearity | Nonlinear vs linear cardiac index | χ²=0.32 | 0.956 |

| **Flow state** | **Ratio group** | **N** | **In-hospital mortality** | **30-day mortality** |
| --- | --- | --- | --- | --- |
| Low <2.2 | All | 850 | 11.8% | 14.9% |
| Low <2.2 | Q2-Q4 | 578 | 9.0% | 10.7% |
| Low <2.2 | Q1 | 272 | 17.6% | 23.9% |
| Normal 2.2-4.0 | All | 1146 | 6.8% | 10.1% |
| Normal 2.2-4.0 | Q2-Q4 | 908 | 5.6% | 8.1% |
| Normal 2.2-4.0 | Q1 | 238 | 11.3% | 17.6% |
| High >4.0 | All | 159 | 10.7% | 15.7% |
| High >4.0 | Q2-Q4 | 129 | 11.6% | 17.1% |
| High >4.0 | Q1 | 30 | 6.7% | 10.0% |

**Table S8. Spline, landmark, and competing-discharge analyses**

| **Outcome** | **Analysis** | **Estimate** | **P** |
| --- | --- | --- | --- |
| In-hospital death after exposure | Ratio spline nonlinearity | χ²=4.50 | 0.212 |
| 30-day death after exposure | Ratio spline nonlinearity | χ²=10.08 | 0.018 |
| In-hospital death after 48-hour landmark | 48-hour landmark logistic model | 1.33 (1.10-1.62) | 0.004 |
| 30-day death after 48-hour landmark | 48-hour landmark logistic model | 1.42 (1.19-1.71) | <0.001 |
| In-hospital death | Cause-specific Cox model (live discharge censored) | 1.26 (1.05-1.51) | 0.012 |

# Supplementary Figures

**Figure S1. Revised cohort flow.**

**Figure S2. Causal diagram used to define the primary adjustment set.**

**Figure S3. Restricted cubic spline for the TAPSE-to-TRPG ratio and in-hospital mortality.**

**Figure S4. Restricted cubic spline for the TAPSE-to-TRPG ratio and 30-day mortality.**

**Figure S5. Competing-risk cumulative incidence with live discharge as a competing event.**
